# A Case of Wolfram Syndrome

**Published:** 2010-01

**Authors:** Gholamali Naderian, Fereshteh Ashtari, Kia Nouri-Mahdavi, Valleh Sajjadi

**Affiliations:** Feiz Hospital, Isfahan University of Medical Sciences, Isfahan, Iran

**Keywords:** Diabetes Mellitus, Diabetes Insipidus, Deafness, Optic Atrophy

## Abstract

**Purpose:**

To report a case of Wolfram syndrome characterized by early onset diabetes mellitus and progressive optic atrophy.

**Case Report:**

A 20-year-old male patient with diabetes mellitus type I presented with best corrected visual acuity of 1/10 in both eyes with correction of −0.25+1.50@55 and −0.25+1.50@131 in his right and left eyes, respectively. Bilateral optic atrophy was evident on fundus examination. The patient also had diabetes insipidus, neurosensory deafness, neurogenic bladder, polyuria and extra-residual voiding indicating atony of the urinary tract, combined with delayed sexual maturity.

**Conclusion:**

One should consider Wolfram syndrome in patients with juvenile onset diabetes mellitus and hearing loss. Ophthalmological examination may disclose optic atrophy; urologic examinations are vital in such patients.

## INTRODUCTION

Wolfram syndrome (WS) is an autosomal recessive neurodegenerative disorder characterized by early onset diabetes mellitus and progressive optic atrophy in the first decade of life.^1^,^2^ This condition is also known as DIDMOAD syndrome, an acronym composed of diabetes insipidus (DI), diabetes mellitus (DM), optic atrophy (OA) and deafness (D).^3^ In 1938, Wolfram et al,^4^ described eight siblings from a family of whom four had diabetes mellitus and optic atrophy. Subsequently three of these four subjects developed sensorineural hearing loss, and two developed a neurogenic bladder. Minimal criteria for establishing the diagnosis are diabetes mellitus and optic atrophy. Diabetes insipidus, sensorineural deafness, urinary tract atony, ataxia, peripheral neuropathy, mental retardation and psychiatric disorders are additional findings present in the majority of patients.^5^

The sequence of the Wolfram syndrome gene (WFS1) was described in 1998 and mutations in this gene have been reported in many populations. To date, the function of the putative protein remains unknown.^5^ A candidate gene has recently been identified on chromosome 4p16.1, but there is evidence of locus heterogeneity and a minority of patients may harbor a mitochondrial genome deletion.^6^

This syndrome should be considered in young diabetic patients with unexplained visual loss or polyuria and polydipsia in the presence of high blood sugar. The exact prevalence of Wolfram syndrome is unknown but a rate of one in 770,000 as estimated in a UK study,^7^ where a physician might see one or two cases in his or her working life time, seems realistic. Although there is a high prevalence of consanguineous marriages in the Middle East and North Africa, only few cases of Wolfram syndrome have been reported from these regions. It may therefore be reasonable to assume that the condition is under-reported from this part of the world.

## CASE REPORT

**Figure 1:**
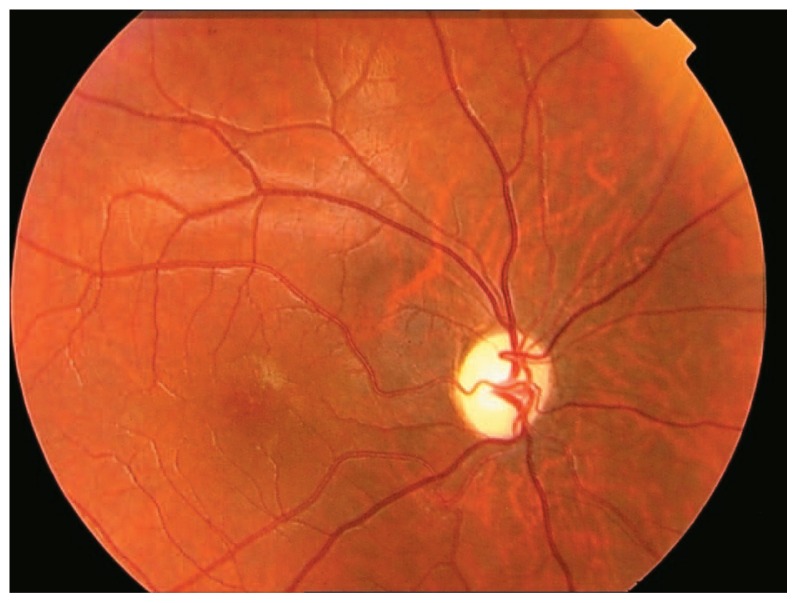
Optic atrophy in the right eye.

**Figure 2:**
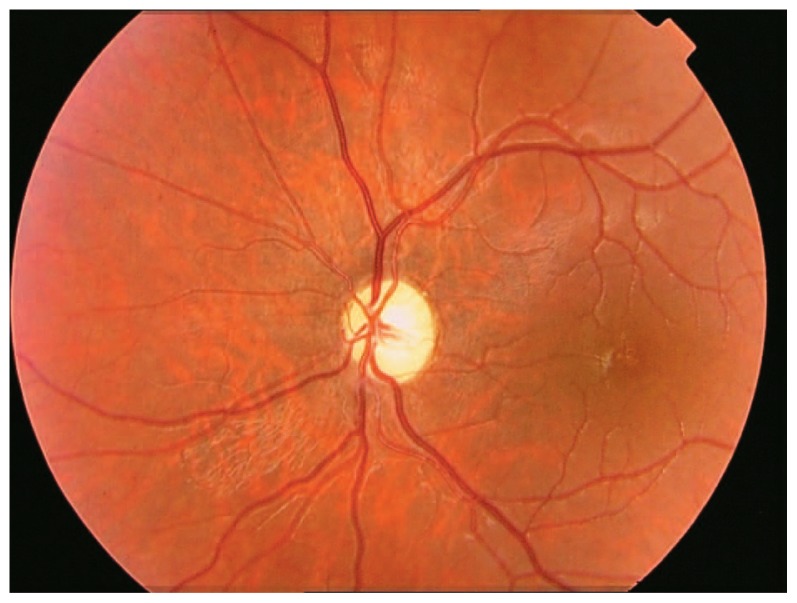
Optic atrophy in the left eye.

**Figure 3:**
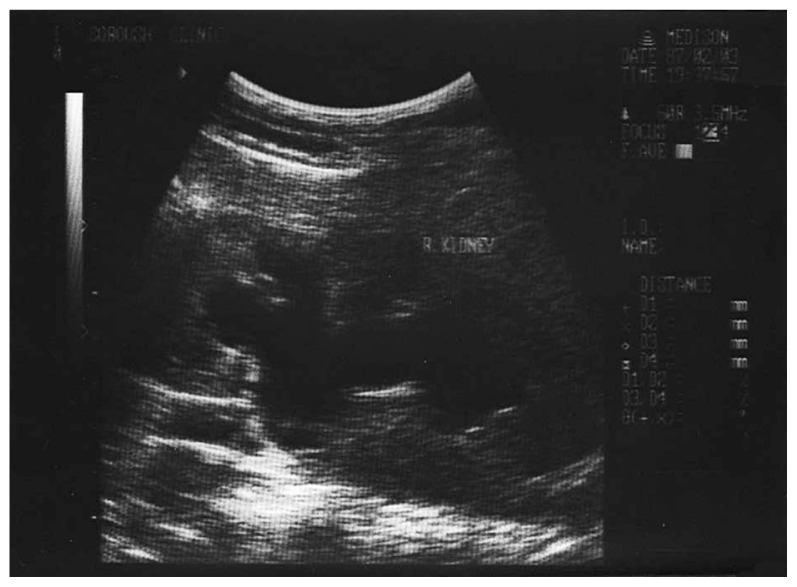
Bladder sonography is consistent with a neuorogenic bladder.

A 20-year-old male patient, a known case of diabetes mellitus type I from 7 years before, presented to a private ophthalmology office. His blood sugar was inadequately controlled even with insulin injections. His parents were consanguineous but healthy, however his grandparents were diabetic. Best-corrected visual acuity was 1/10 in both eyes with −0.25+1.50@55 and −0.25+1.50@131 in his right and left eyes respectively. Intraocular pressure was 14 mmHg in both eyes and biomicroscopic examinations were completely normal. Fundus examination showed advanced bilateral optic atrophy but no sign of diabetic retinopathy (Figures 1, 2). Laboratory studies and dehydration test confirmed diabetes insipidus. An audiologic examination revealed neurosensory deafness. Magnetic resonance imaging (MRI) of the brain and lumbar spine was normal. Both kidneys were larger than normal and showed signs of moderate hydronephrosis on ultrasonographic evaluation. The bladder wall had a trabecular pattern and the urinary tract was dilated on both sides. The patient had 170 ml post-voiding bladder residue. Urologic examination and sonographic findings were consistent with a neurogenic bladder (Figure 3). The patient also had polyuria and extra-residual voiding which indicated atony of the urinary tract. Urogenital examination revealed some degree of delayed sexual maturation.

## DISCUSSION

Wolfram syndrome is a progressive autosomal recessive neurodegenerative disorder.^1^ Hallmarks of the syndrome are diabetes mellitus, which is usually the first sign of the disease (median age at diagnosis, 6–15 years), and optic atrophy (median age at diagnosis 11 years). Optic atrophy in a diabetic patient necessitates audiometry and intravenous pyelography.^8^ In our patient the majority of symptoms became manifest by the second decade of life and a correct diagnosis had been made when he was 15 years old.

Hearing loss, mainly in high frequencies, may be present in 48% of patients and diabetes insipidus of hypothalamic origin may occur in the third decade of life in up to 75% of cases.^9^,^10^ Dilation of the urinary tract is observed in 45% of cases which may be secondary to chronic high urine flow rates (diabetes insipidus) or neuronal degeneration at various levels of the urinary tract.^11^ Atony of the bladder and the whole urinary tract may also be observed. Additionally, retinal pigmentary changes, spinocerebellar degeneration, delayed sexual maturation, a small sella turcica and male hypogonadism due to primary gonadal failure have been reported with DIDMOAD syndrome.^12^–^14^

Differential diagnoses include congenital rubella syndrome, Leber’s hereditary optic atrophy, and thiamine responsive anemia with diabetes mellitus and deafness. The association of diabetes mellitus with optic atrophy also occurs in Friedreich’s ataxia, Refsum disease, Alstrom syndrome, Lawrence-Moon syndrome, Kearn-Sayre syndrome, and deafness and diabetes in the “3243” mitochondrial DNA mutation.^8^

The pathogenesis of the disease remains unknown, but positional cloning studies in families with Wolfram syndrome have identified linkage peaks on the short arm of chromosome 4 (4p16.1). Mutations in the gene encoding wolframin (WFS1), which maps to that region, have also been shown to cause the syndrome.^15^,^16^ Nevertheless, the wide spectrum of clinical manifestations affecting several organs and tissues, suggests mitochondrial DNA (mtDNA) involvement.

Since the initial identification of the *WFS1* gene by Inoue et al,^17^,^18^ different research groups have reported more than 50 distinct mutations in this gene. WFS1 protein presumably functions to maintain certain populations of neuronal and endocrine origin. Diabetes mellitus may result from hypothalamic degeneration, although loss of pancreatic β-islet cells as part of a specific defect in neuroectodermal amine precursor uptake and decarboxylation-derived cells in the pancreas and in the supraoptic and paraventricular nuclei has also been postulated. DI is thought to be related to atrophy and degeneration of the hypothalamus with loss of vasopressin-secreting neurons in the supraoptic and paraventricular nuclei, and degeneration of the posterior pituitary gland. The deafness is sensorineural and degenerative; atrophy of the vestibulocochlear nuclei and inferior colliculi may be responsible.^19^

Due to variability of symptoms, Wolfram syndrome may be overlooked. The condition should be evaluated in a multidisciplinary manner and specific tests are necessary to make a precise diagnosis and disclose all components of the syndrome. Management requires cooperation between several specialists including an endocrinologist, neurologist, ophthalmologist and urologist.
